# Investigating the Role of Culture on Negative Emotion Expressions in the Wild

**DOI:** 10.3389/fnint.2021.699667

**Published:** 2021-12-08

**Authors:** Emma Hughson, Roya Javadi, James Thompson, Angelica Lim

**Affiliations:** Robots With Social Intelligence and Empathy Lab, Simon Fraser University, School of Computing Science, Burnaby, BC, Canada

**Keywords:** affective computing, negative emotions, emotion recognition, emotions, culture studies, facial expressions, dynamic social signals, machine learning

## Abstract

Even though culture has been found to play some role in negative emotion expression, affective computing research primarily takes on a basic emotion approach when analyzing social signals for automatic emotion recognition technologies. Furthermore, automatic negative emotion recognition systems still train data that originates primarily from North America and contains a majority of Caucasian training samples. As such, the current study aims to address this problem by analyzing what the differences are of the underlying social signals by leveraging machine learning models to classify 3 negative emotions, contempt, anger and disgust (CAD) amongst 3 different cultures: North American, Persian, and Filipino. Using a curated data set compiled from YouTube videos, a support vector machine (SVM) was used to predict negative emotions amongst differing cultures. In addition a one-way ANOVA was used to analyse the differences that exist between each culture group in-terms of level of activation of underlying social signal. Our results not only highlighted the significant differences in the associated social signals that were activated for each culture, but also indicated the specific underlying social signals that differ in our cross-cultural data sets. Furthermore, the automatic classification methods showed North American expressions of CAD to be well-recognized, while Filipino and Persian expressions were recognized at near chance levels.

## 1. Introduction

Culture has been identified to play a significant role in our upbringing, influencing the perception and expression of emotional experiences (Mesquita et al., 2016; Barrett et al., 2019; Schouten et al., 2020). Culture embeds in humans a set of rules to help select the appropriate display of emotions to navigate social environments in one's culture (Ekman and Friesen, 1971). In the area of affective computing, culture has had some exploration investigating emotion recognition technologies role in mental health cross-culturally (Ringeval et al., 2018, 2019). Nevertheless, there is still a large body of research regarding emotion recognition technologies that treats all cultures equally using a basic emotion, or common view, approach. This approach emphasizes a common underlying structure for each emotion expressed cross-culturally (Barrett et al., 2019). On the other hand, a social constructivist approach emphasizes individuality of social influence on humans emotion expression and perception (Fragopanagos and Taylor, 2005). Furthermore, researchers, such as Jack et al. (2012) have illustrated that different cultures demonstrate emotions differently. Taking on a basic-emotion approach could impact the performance of emotion recognition technologies by treating training data that originates from different cultures, equally. Furthermore, current data sets primarily contain Caucasian and North American training samples or do not categorize by culture by incorporating culture tags during data set generation (Feng and Chaspari, 2020). Therefore, these available data sets potentially increase algorithm bias, hindering the inclusion of all cultures (Sharma and Dhall, 2020). Including cultural categories could increase model robustness, providing a more versatile culturally-aware emotion recognition system.

Historically, researchers have found an implicit ability in humans to detect emotions with higher accuracy when interacting with someone who is in their cultural group (in-group advantage) (Ekman, 1971; Elfenbein and Ambady, 2002). Culture can be broken down into individualistic and collectivist subsets which can be ranked using Hoftsede's Individualism (IDV) score by placing cultures on a scale between 1 (purely collectivist) and 100 (purely individualistic) (Hofstede, 2007). Individualism characterizes individuals who are loosely tied to others, prioritizing themselves along with immediate family. On the other hand, collectivism characterizes individuals who are tightly linked with others, prioritizing the well-being of the group over themselves. Cultures who have low IDV scores have shown to suppress negative emotions to maintain harmony in social interaction, whereas cultures with high IDV scores idealize self-expression and open communication (Fernandez et al., 2000; Hofstede, 2007; Schouten et al., 2020). One study assessed social robots' cultural sensitivity by extracting facial Action Units (AUs) from East Asian culture, as the definition of universality of facial expressions was more tailored to Western cultures (Chen et al., 2019). East Asian participants identified the culturally derived expressions with higher recognition accuracy compared to the existing set of expressions that were placed on a robot's face, indicating that there is also cultural discrepancy regarding how one expresses and perceives emotions. In addition, Jack et al. (2012) found significant differences in the actual activation of facial Action Units (AUs) when mapping the intensity of each AU across Western Caucasian and East Asian cultures.

Most affective computing research regarding emotion recognition technologies focus on 7 distinct emotions (i.e., happiness, sadness, anger, disgust, contempt, fear, and surprise) (Liliana and Basaruddin, 2018; Sergeeva et al., 2019). There is a tendency to investigate all seven emotions together instead of separating them into positive and negative groups. Although covering more ground by including all emotion categories, these studies can become too broad and overshadow underlying differences of negative emotions. Furthermore, negative emotion recognition in both technology and humans has been found to have the worst accuracy discrepancy when performance is compared across different cultures. For example, Chen et al. (2019) reviewed 15 different studies and found that overall human participants from western cultures were able to recognize fear, disgust, and anger with high accuracy, whereas other cultures' recognition was much lower. Siqueira et al. (2020) found that using the popular AffectNet data set, resulted in accuracy between 45 and 54% for contempt, anger, and disgust, while happiness had 77%. In addition, contempt is also usually the worst performing negative emotion. When using the FER+ data set, Siqueira et al. (2020) found contempt could only reach 20% accuracy, whereas happiness reached 95%. Vo et al. (2020) also found that not only did contempt perform the worst, with 23% accuracy, but also that it had fewer examples available in emotion data sets. Although images themselves can be used for classification purposes (e.g., using a convolutional neural network) the current study aims to address how AUs vary amongst different cultures and how using AUs as attributes can be used to classify negative emotions.

For the current study, we focused on the emotions of contempt, anger and disgust (the so-called CAD Triad, Rozin et al., 1999) across three different cultures that range from highly individualistic to highly collectivist. As previously mentioned, negative emotions, in particular contempt, have been hard to predict cross-culturally in both humans and emotion recognition technologies (Siqueira et al., 2020; Vo et al., 2020). According to the CAD Triad model, each of the mentioned emotions is evoked when one of the three moral codes are violated. Contempt is elicited when community codes are violated, anger is elicited when individual rights are violated, and disgust is elicited when divinity codes are violated causing impurity against oneself, others or God (Rozin et al., 1999). In the context of the current study, negative emotions will be broken down into their social signals, or AUs. AUs map facial landmarks that are activated when a given emotion is expressed (Yang et al., 2007; Lucey et al., 2011). Disgust, described as a wrinkled nose and mouth, is associated with the activation of AU4 (brow lowerer), AU9 (nose wrinkler), AU10 (upper lip raiser), AU17 (chin raiser), and AU 20 (lip stretcher) (Scherer et al., 2019; Singh et al., 2020). Anger, described as protruding teeth and tightened eyes looking downward, is associated with the activation of AU4 (brow lowerer), AU5 (upper lid raiser), and AU27 (mouth stretch). Contempt involves a raised and tightened unilateral lip corner and is associated with the activation of AU4 (brow lowerer), AU7 (lid tightener), AU10 (upper lip raiser), AU25 (lips part), and AU26 (jaw drop) (Matsumoto and Ekman, 2004; Scherer et al., 2019; Singh et al., 2020). Using the associated AUs for each emotion we can map their descriptive qualities (e.g., activation of AU9 can be mapped to the wrinkled nose description of disgust). Yet these basic emotion expression templates do not explain the variance of expressions in the wild (Barrett et al., 2019). We aim to address the literary gap regarding culture and emotion recognition technology by using a data-driven analysis of different AUs and their level of activation to investigate culture's influence on social signals associated with negative emotion expression.

## 2. Materials and Methods

### 2.1. Data Set and Features

Our data set consists of 257 short video clips (between 1 and 11 s) collected from YouTube that depicted contempt, anger, and disgust (the CAD Triad) across Canadian and American (High Individualism Score) cultures, along with Persian (Medium Individualism Score) and Filipino (Low Individualism Score) cultures. The Canadian and American groups were categorized together and considered as one culture called North American culture. Video clips in each culture were collected and annotated by a three data collectors, one from each culture. Six volunteers in addition to the three data collectors, two from each culture category, added additional annotation for the clips in their identified culture. A clip was labeled with a given emotion label where at least two of the three annotators agreed on the given label, therefore establishing ground truth for each clip. If no annotators agreed on a given label then that clip was held-out of the experiment, as ground truth for that clip could not be established. These in-the-wild clips were taken from either professionally acted (movies) or spontaneous (reality TV, vlogs) scenarios. Filipino culture contained 74 videos displaying either anger (25), contempt (30), or disgust (19). Persian culture contained 75 videos displaying either anger (27), contempt (28), or disgust (20). Finally, North American culture contained 108 videos displaying either anger (48), contempt (39), or disgust (21). The entire data set is over a total of 15 min and contains 27,020 frames.

**Figure 1:**
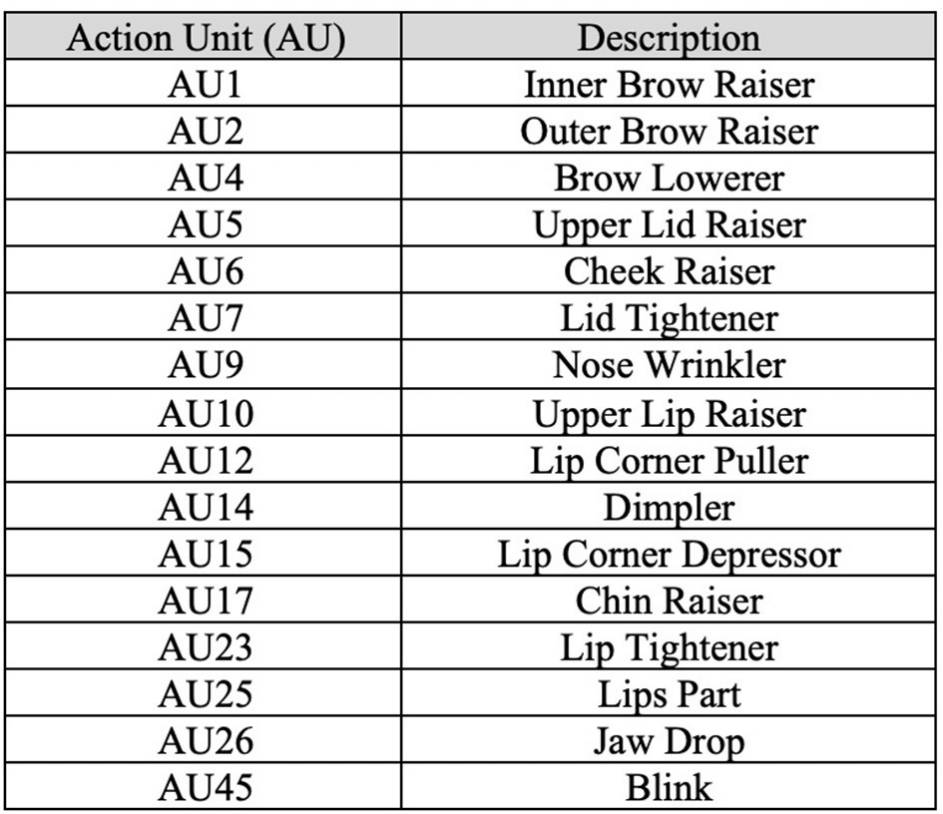
Collected AUs with their associated definitions.

After video collection, OpenFace (Baltrusaitis et al., 2018) was used to extract social signals (i.e., activation levels of AUs) for each frame in a given video. Only 17 AU attributes were collected (i.e., AU1, AU2, AU4, AU5, AU6, AU7, AU9, AU10, AU12, AU14, AU15, AU17, AU23, AU25, AU26, and AU45) describing relative values for each AU. Refer to Figure 1, which contains AUs and their associated definitions. Relative values measure the amount of activation for each AU ranging from 0 to 5 (e.g., high values indicate high activation). Confidence level and success level, which indicate how confident the software is that a given frame successfully exhibited a given set of AUs and the associated level of activation, were also collected. For each frame the originating filename, frame number, and culture were noted indicating origin of each data point in our data set.

During preprocessing, extra features produced by OpenFace, such as head movement, head position, and gaze direction, were removed. Furthermore, we only chose the frames that had a high confidence level, above 80%, that a given AU was activated. Frames with success that was less than 1 were also removed. In addition, if a clip contained multiple subjects, data for the subjects not displaying the emotions was discarded. Since the available clips for each emotion in each culture had varying amounts, the training data set for a given emotion category was balanced by randomly removing videos to make each emotion category have the same amount of available training data within a given culture. In addition, the smallest available data set (disgust) in each culture is approximately 20 clips. Thus each culture and emotion category was trained on a similar amount of clips in order to remove bias toward one culture or one emotion. For example, the North American data set had clips removed randomly for emotion categories contempt and anger, to ensure that they contained 21 video clips, the same as the smallest emotion category, disgust.

### 2.2. SVM Model for Classification

Establishing a robust machine learning model to classify emotions is important as it can greatly impact performance outcomes. Swinkels et al. (2017) used a cascade of both a binary and multi-class support vector machine (SVM) to classify emotions. They found that using an SVM for classification outperformed state-of-the-art algorithms, specifically when detecting contempt and disgust. Others have also found success using an SVM in comparison to other commonly used classification methods when recognizing different emotions in older adults and children (Nojavanasghari et al., 2016; Ma et al., 2019). There has also been a rise in research that uses SVM in conjunction with deep learning models. For example, Muhammed et al. (2020) used a convolutional neural network (CNN) to extract features in images and then used a SVM to classify images based on the extracted features with 70–85% accuracy. Although results using a CNN with a SVM are good, Baltrusaitis et al. (2018) found even more promise using a Convolutional Experts Constrained Local Model (CE-CLM) for the toolkit OpenFace, beating state-of-the-art methods in regards to extracting AUs. Furthermore, given the current study is exploring the differences in the underlying expressions of negative emotions, the additional implementation of a deep learning pipeline could reduce interpretability, as feature embeddings are not as visualizable as activation levels of AUs. As such, OpenFace was used to extract the activation levels for the various AUs in addition to using a SVM for classification, given its previous success on negative emotion recognition.

A SVM uses binary classification to separate training vectors along with class labels into two categories using decision boundaries (Huang et al., 2017). The training vectors are *n*-dimensional and in our case we have 16 AUs, therefore, *n* is 16. The category labels are emotion categories. The SVM then maps the input vectors onto a higher-dimensional feature space. The goal is to optimize the distance between the line that separates the two categories, called a hyperplane, and support vectors that indicate points that are closest to that line. Therefore, optimizing our decision boundary and increasing the separation of categories. The hyperplane is constructed using a kernal function. In the current study, the kernal function is linear and the cost *c* = 1.8, as it introduced the least amount of error during cross-validation. While the linear kernel had the best performance on this data set, other work on detecting emotions in still images showed better accuracy using quadratic kernels (Adeyanju et al., 2015). The SVM and k-fold cross-validation was implemented using an open source python library called Scikit-learn (Pedregosa et al., 2011).

### 2.3. Experiments

The current study addresses two hypotheses: (1) cultures express the emotions of the CAD Triad differently, and (2) the CAD Triad cannot be predicted using machine learning models across three different cultures when considering all cultures equivalently.

#### 2.3.1. Differing Expressions of CAD Triad

We first hypothesize that for a given CAD emotion, AU activation will be different for each culture, indicating that social signals used to display the CAD triad are not universal. To investigate this hypothesis, each AU related to an emotional category (i.e., contempt, anger, and disgust) will be analyzed using a one-way ANOVA. The AUs associated with each emotion will be compared cross-culturally to identify if there is a significant difference in means of AU activation across each culture. Furthermore, a heat map, or activation map, will be created using all AUs for each culture to identify what AUs, with a activation level threshold of at least 2.5, are highly activated for each emotion. Differences in activation maps would further support the premise that AUs activated for each culture are different.

#### 2.3.2. Within-Culture and Cross-Cultural Recognition of CAD Triad

Our second hypothesis is the CAD Triad cannot be predicted cross-culturally and as such, model performance will be better when cultures are considered separately. We will test our second hypothesis with two experiments, examining: (1) the accuracy when performing within-culture training and testing, and (2) the accuracy when performing cross-cultural training and testing. Both experiments will include each of the three culture data sets: North American, Persian, and Filipino. Both experiments use a SVM as a training and prediction model. Since the data set is relatively small, a five-fold cross-validation process will be used. Before feeding data into the two models, around 25% of the clips will be randomly held out for testing to assess the models ability to generalize. Accuracy and f1-score for each fold will also be calculated and averaged over all folds as an evaluation metric. Both experiment will use prediction performance (i.e., accuracy and f1-scores) to compare cultural categories.

The goal of the first experiment is to assess the model's recognition performance of each culture independently, by training an SVM on one culture and testing on the same culture. The comparison between cultures will provide a baseline to assess how well the model can predict using our feature set, independently of other cultures. This experiment will also provide information on the similarity of CAD within a given culture. The second experiment will be to train an SVM on one of the three culture data sets and test on one of the remaining two cultures. The accuracy for each training and testing combination will be compared with one another. We expect that within-culture accuracy will be higher than cross-culture accuracy, indicating a in-group advantage.

## 3. Results

### 3.1. Statistical Analysis

**Figure 2:**
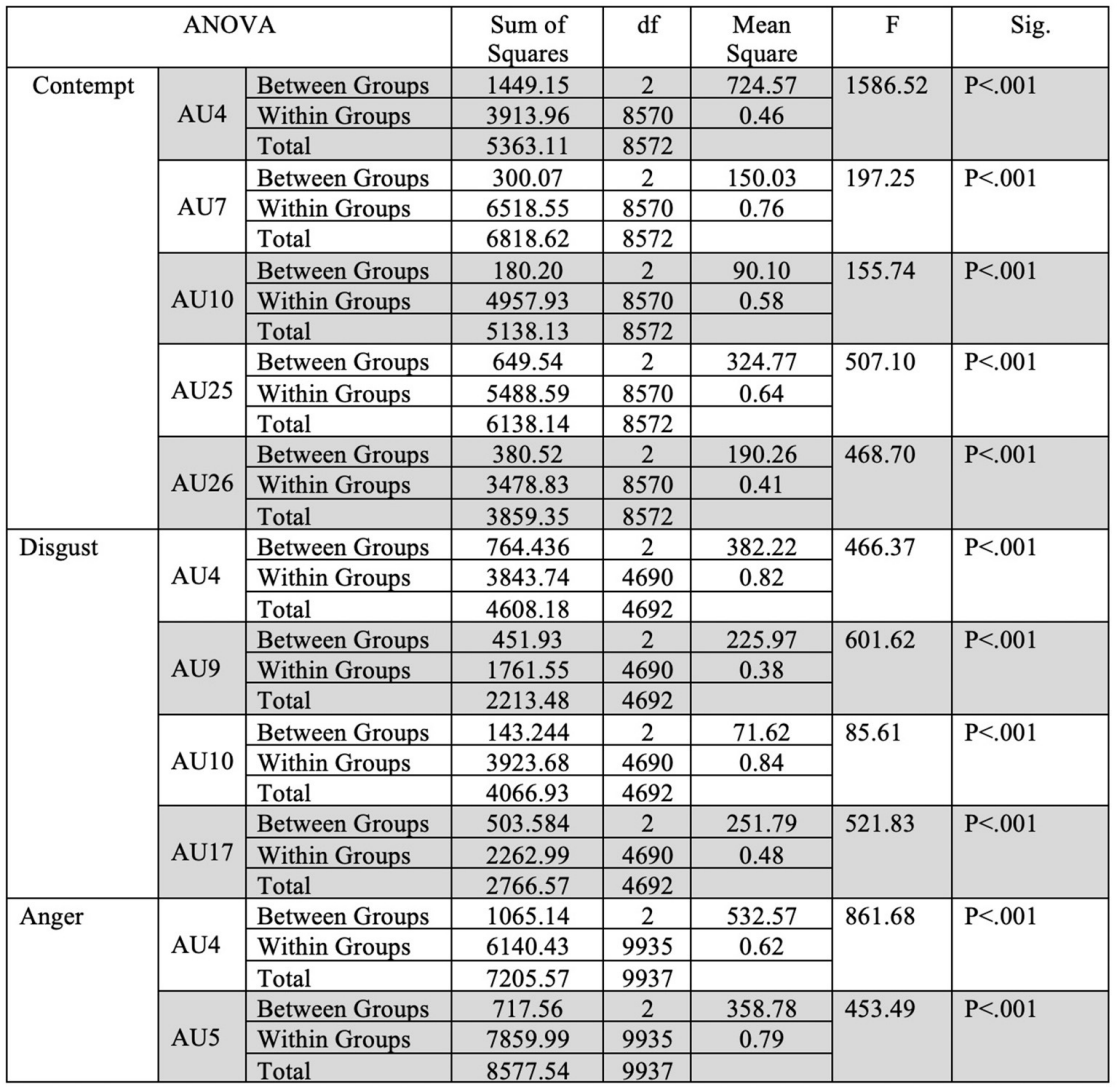
Descriptive statistics for the one-way ANOVA for each emotion across all cultures.

**Figure 3:**
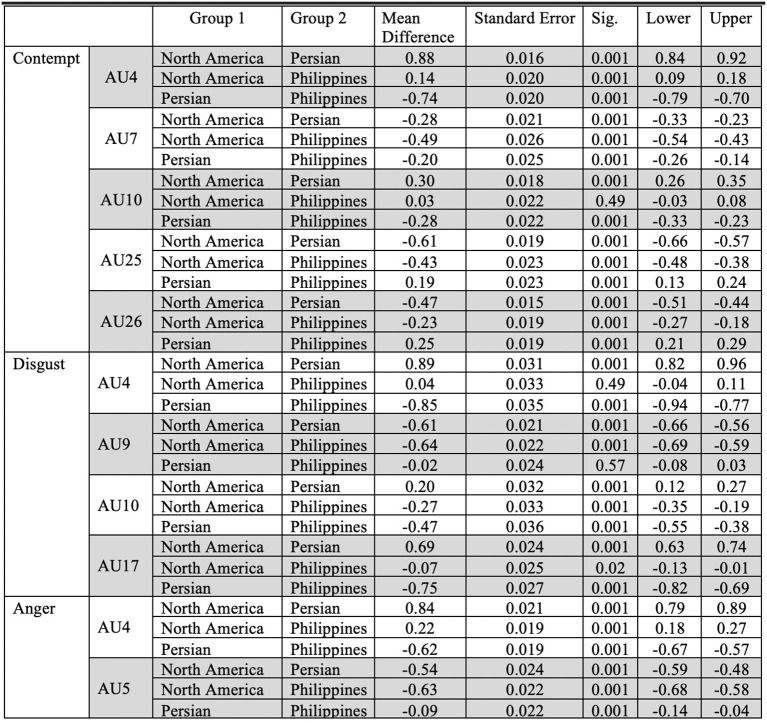
Descriptive statistics for the *post-hoc* Tukey Test for each emotion across all cultures.

**Figure 4:**
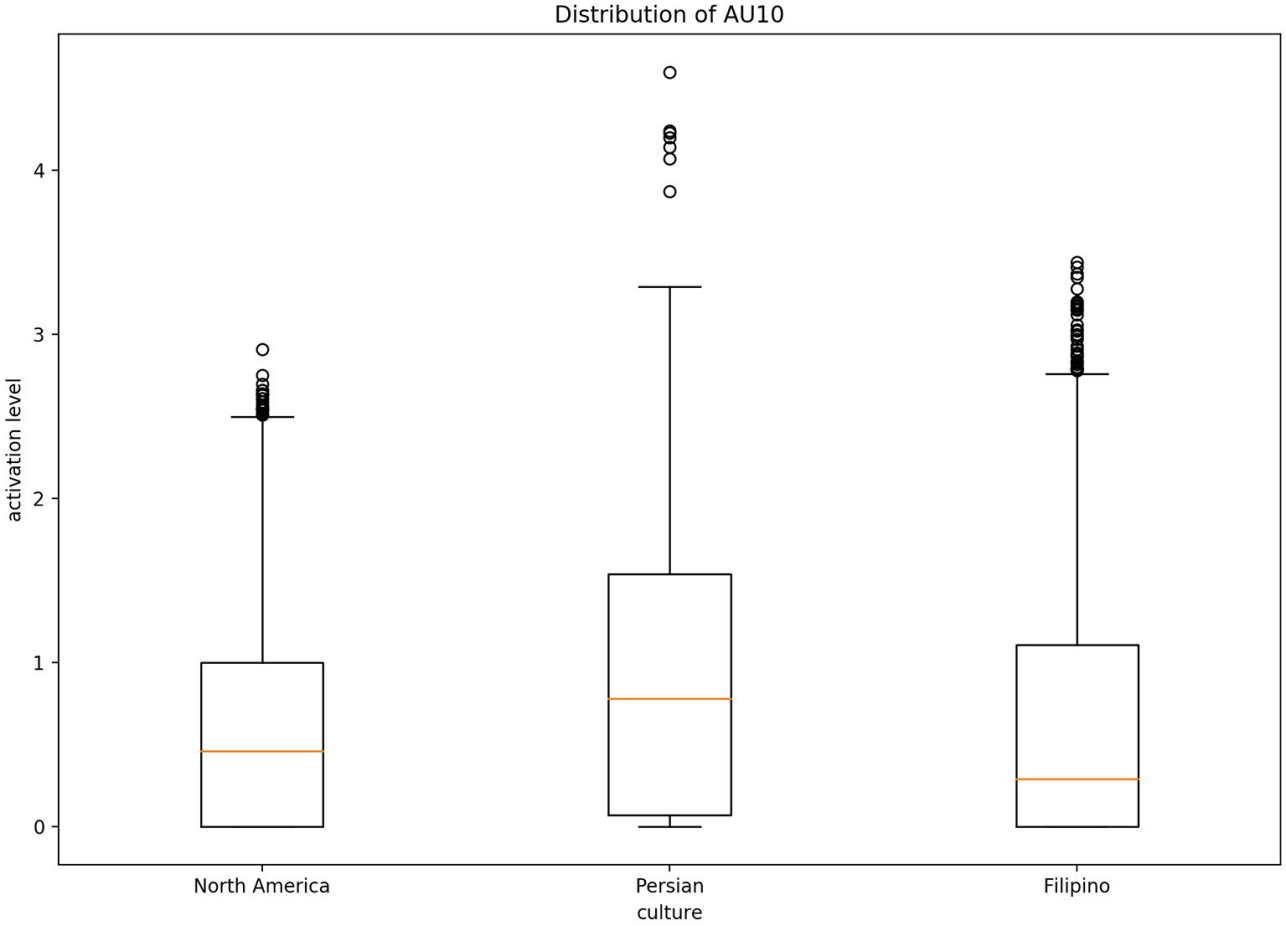
Distribution of AU10 cross-culturally for contempt.

**Figure 5:**
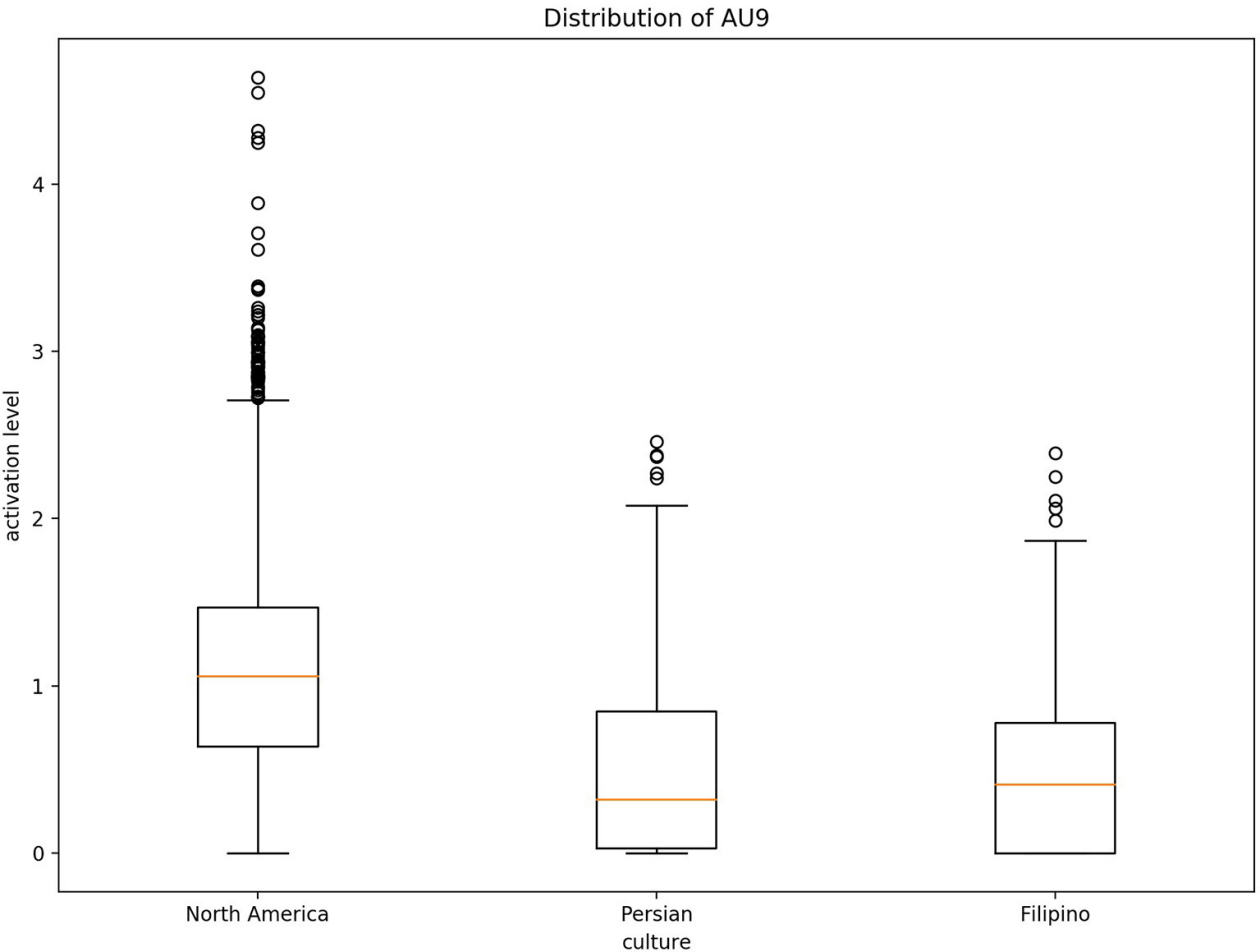
Distribution of AU9 cross-culturally for disgust.

**Figure 6:**
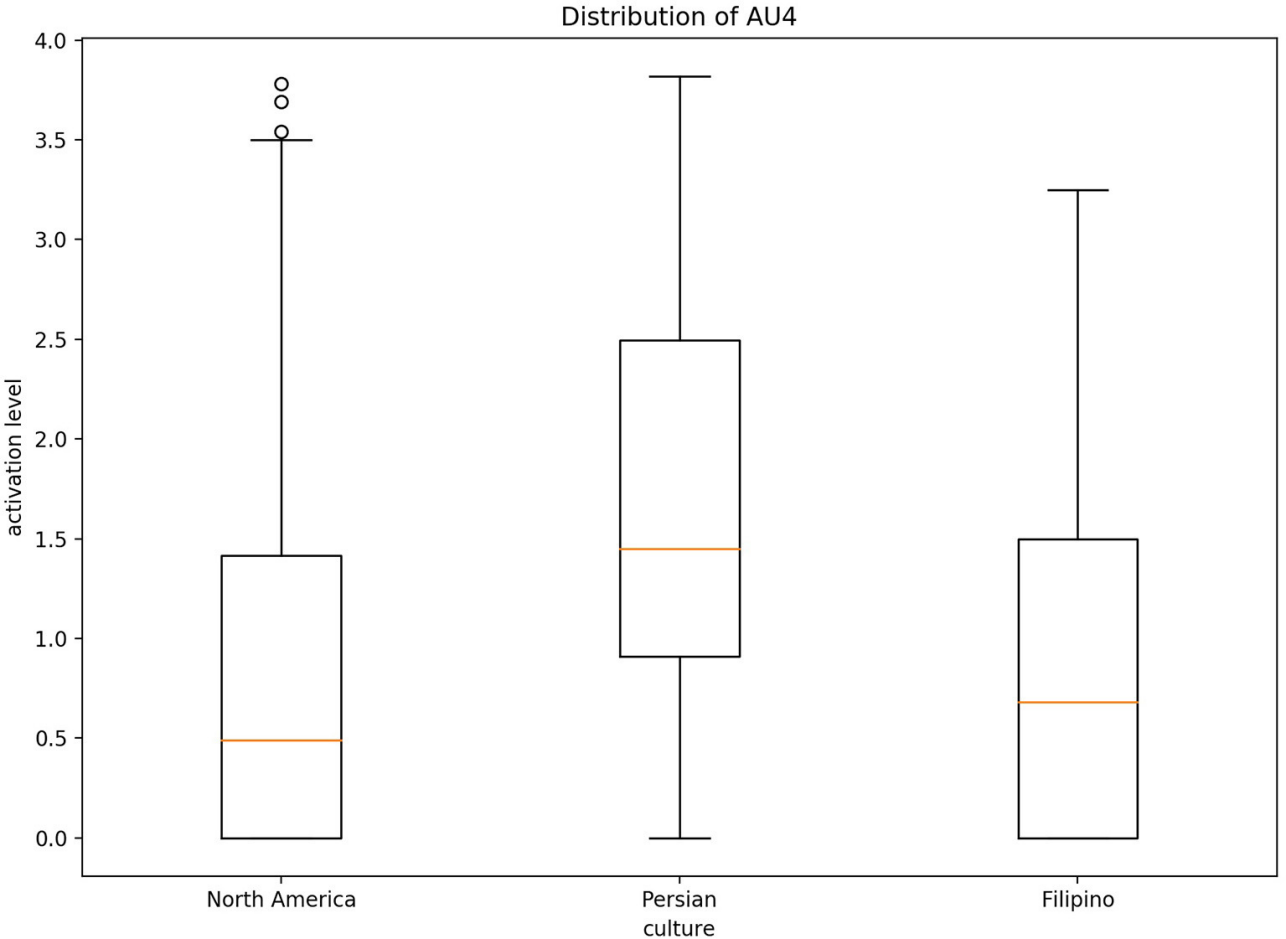
Distribution of AU4 cross-culturally for disgust.

Fleiss' Kappa Inter-rater agreement was 0.83 for the North American data set, 0.40 for the Filipino data set, and 0.59 for Persian data set. An one-way ANOVA (Figure 2) was conducted on each emotion category across each culture category to compare the effect of culture on each emotions' associated level of AU activation. For contempt, analysis of variance demonstrated that cultures effect on AU4 [*F*_(2, 8,570)_ = 1586.35, *p* < 0.05], AU7 [*F*_(2, 8,570)_ = 197.25, *p* < 0.05], AU10 [*F*_(2, 8,570)_ = 155.74, *p* < 0.05], AU25 [*F*_(2, 8,570)_ = 507.10, *p* < 0.05], and AU26 [*F*_(2, 8,570)_ = 468.70, *p* < 0.05], were statistically significant. A *Post-hoc* Tukey Test (Figure 3) indicated that each cultural group differed significantly in activation for each AU, *p* < 0.05, except AU10. AU10 did not significantly differ on North American and Filipino data sets (Figure 4). For assessing cultures effect on anger, a one-way ANOVA was applied to AU4 and AU5 independently. An analysis of variance on AU4 and AU5 resulted in significant variation amongst culture, *F*_(2, 9,935)_ = 861.68, *p* < 0.05; *F*_(2, 9,935)_ = 453.49, *p* < 0.05. *Post-hoc* Tukey test also indicated that each cultural group differed significantly, *p* < 0.05. Finally, to assess disgust, a one-way ANOVA was applied to AU4, AU9, AU10, and AU17 independently. An analysis of variance on AU4 [*F*_(2, 4,690)_ = 466.37, *p* < 0.05], AU9 [*F*_(2, 4,690)_ = 601.62, *p* < 0.05], AU10 [*F*_(2, 4,690)_ = 85.61, *p* < 0.05], and AU17 [*F*_(2, 4,690)_ = 521.83, *p* < 0.05] resulted in statistically significant variation amongst cultures. A *Post-hoc* Tukey test also confirmed the statistical significance for each AU and culture, *p* < 0.05. Except, AU9 and AU4 did not have a statistically significant result between the Persian data set and the Filipino data set (Figure 5) and the North American and the Filipino data set (Figure 6), respectively. It is important to note that some AUs were not available for collection or could not be reliably detected using OpenFace, such as AU20 and AU27 which are associated with disgust and anger, respectively.

### 3.2. Activation Map: Culture-Specific Social Signals of CAD Triad

**Figure 7:**
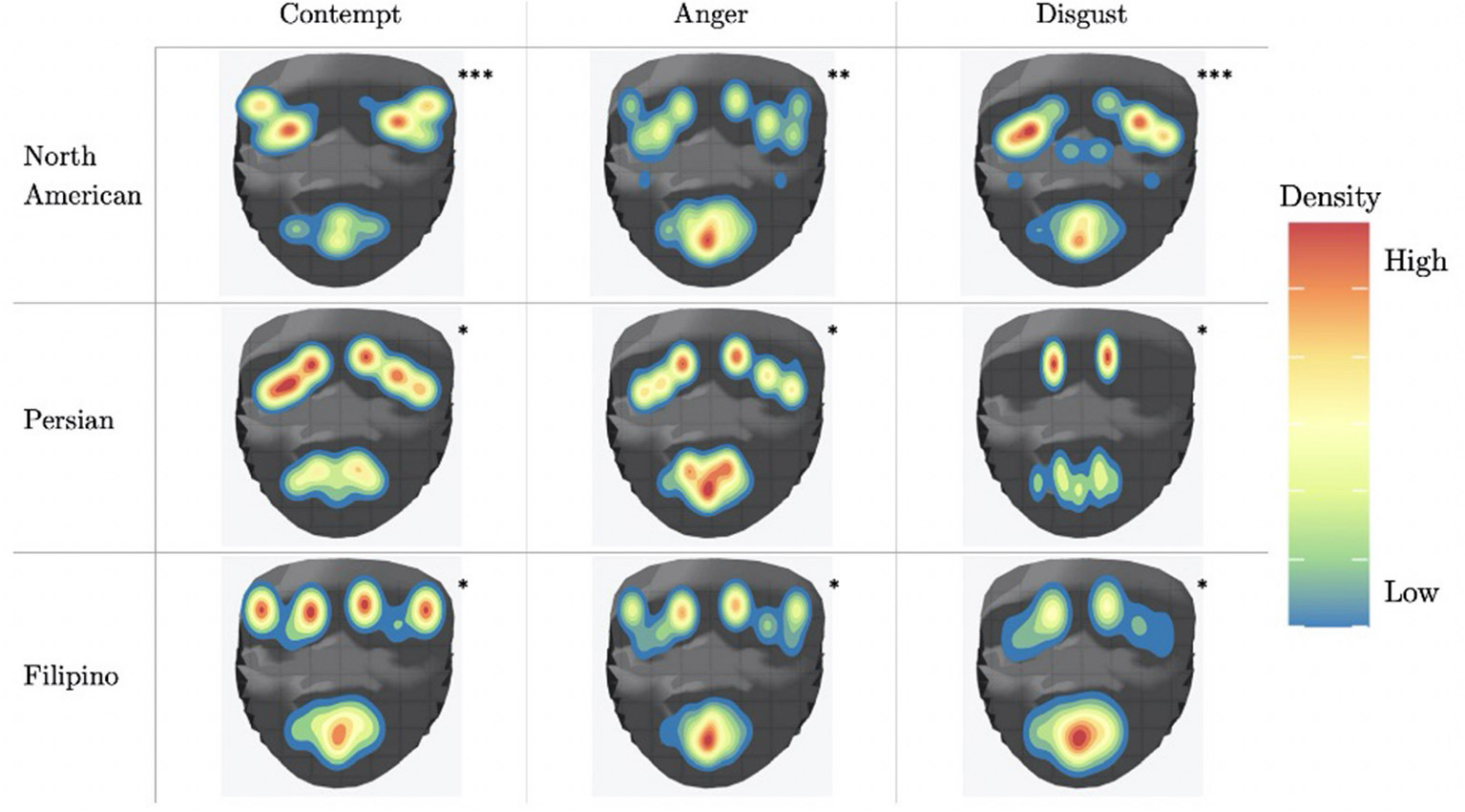
AU activation map that shows the localization of activation using a threshold of 2.5. ^*^Movie/TV shows as primary source; ^**^Mix of Movie/TV shows, Reality TV and Vlogs as primary source; ^***^Reality TV and Vlogs as primary source.

In addition to conducting a one-way ANOVA, an activation map, which is shown in Figure 7, was used to illustrate the activation of all AU's in the data sets for each culture. Each action unit is mapped to one or more 2-dimension facial points as proposed by Ghayoumi and Bansal (2016). A threshold of at least 2.5 selects action units that are activate. To simulate the facial movement captured by action units, we added random variance in the direction of movement and a small amount of noise to produce smoother density plots. The activation map indicates which action units are more likely to activated for a given culture and emotion as compared to other action units within that same culture and emotion.

### 3.3. Data Analysis

#### 3.3.1. Contempt

North American contempt shows AU25 (lips part), AU2 (outer brow raiser), and AU45 (blink), AU7 (lid tightener) and AU12 (lip corner puller) to be highly activated. Persian contempt shows the AU's normally found to be activated with contempt, AU4 (brow lowerer) and AU10 (upper lip raiser), AU1 (inner brow raiser), AU15 (lip corner depressor) and AU25 (lips part). Filipino contempt has both AU1 (inner brow raiser), AU2 (outer brow raiser), and AU25 (lips part) highly activated.

#### 3.3.2. Anger

North American anger is reflected by AU5 (Upper Lid Raiser), AU1 (Inner Brow Raiser), AU25 (lips part) and AU10 (upper lip raiser). Persian anger has AU4 (brow lowerer), AU10 (upper lip raiser), AU1 (inner brow raiser) and AU25 (lips part) highly activated. In terms of Anger, Filipino anger shows high activation of AU1 (inner brow raiser), AU25 (lips part), AU4 (brow lowerer), AU10 (upper lip raiser), and AU26 (jaw drop).

#### 3.3.3. Disgust

North American disgust AU7 (lid tightener), AU25 (lips part), AU9 (nose wrinkler), and AU10 (upper lip raiser). Persian disgust has AU4 (brow lowerer), AU10 (upper lip raiser), AU1 (inner brow raiser), AU15 (lip corner depressor) and AU17 (chin raiser). Finally, Filipino disgust has AU4 (brow lowerer), AU10 (upper lip raiser), AU1 (inner brow raiser), AU26 (jaw drop), AU25 (lips part), AU7 (lid tightener).

### 3.4. Experimental Results

#### 3.4.1. Within-Culture Testing Accuracy

**Figure 8:**
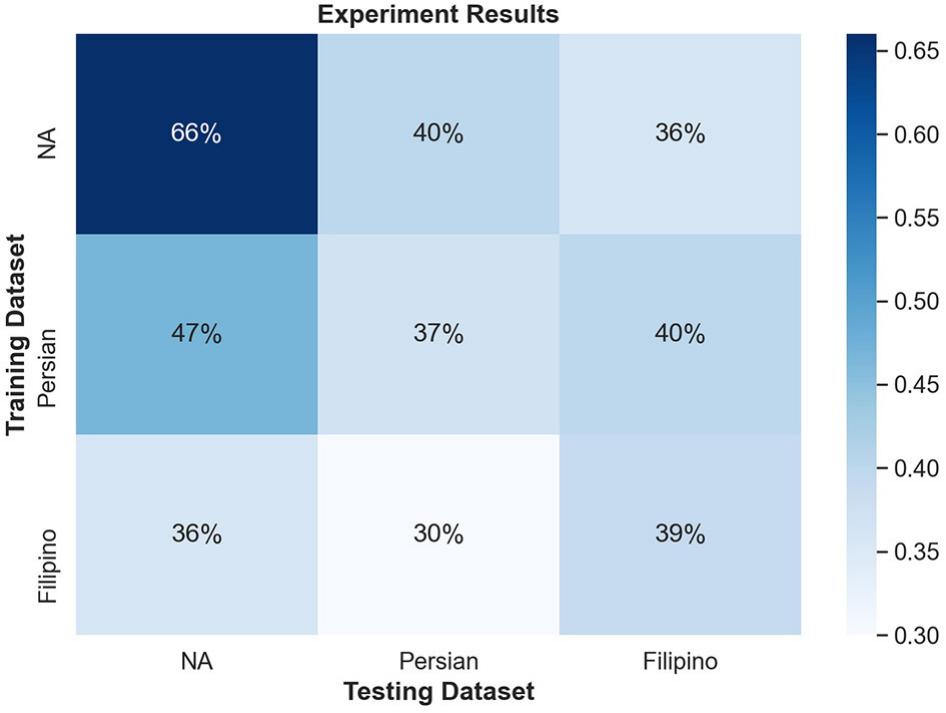
Confusion matrix illustrating the results of all within-culture experiments.

Figure 8 shows the confusion matrix for within-culture experiments for the North American data set. Testing accuracy (i.e., percentage of correct emotion labels) for the North American data set was the highest with 66% accuracy. The next highest testing accuracy was the Filipino data set, with 39%, followed by the Persian data set with 37%.

#### 3.4.2. Within-Culture Confusions

**Figure 9:**
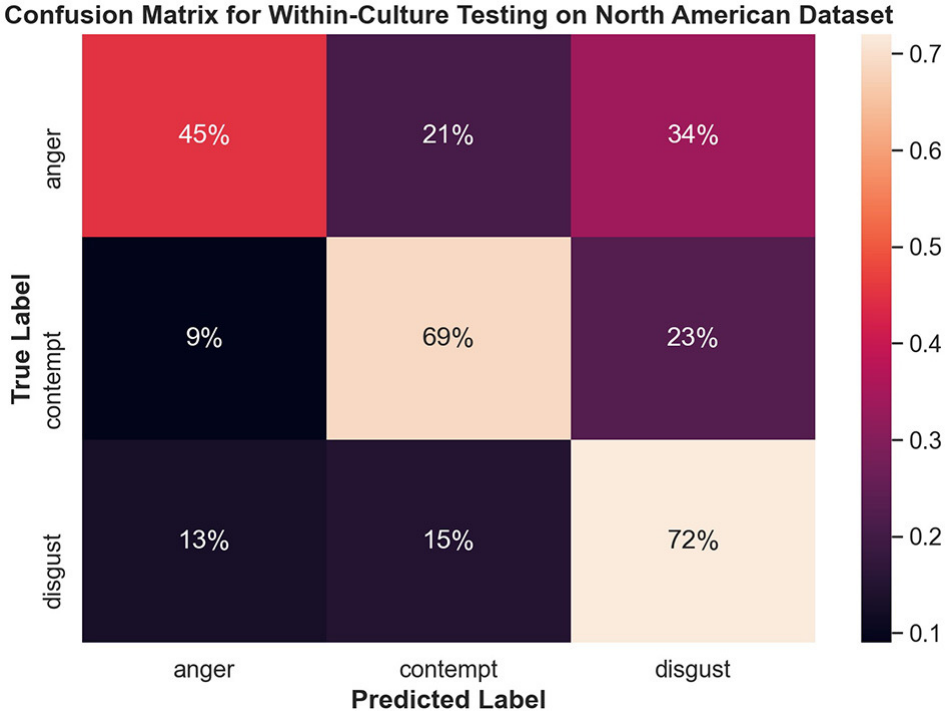
Confusion matrix illustrating the results of the within-culture experiment on the North American data set.

**Figure 10:**
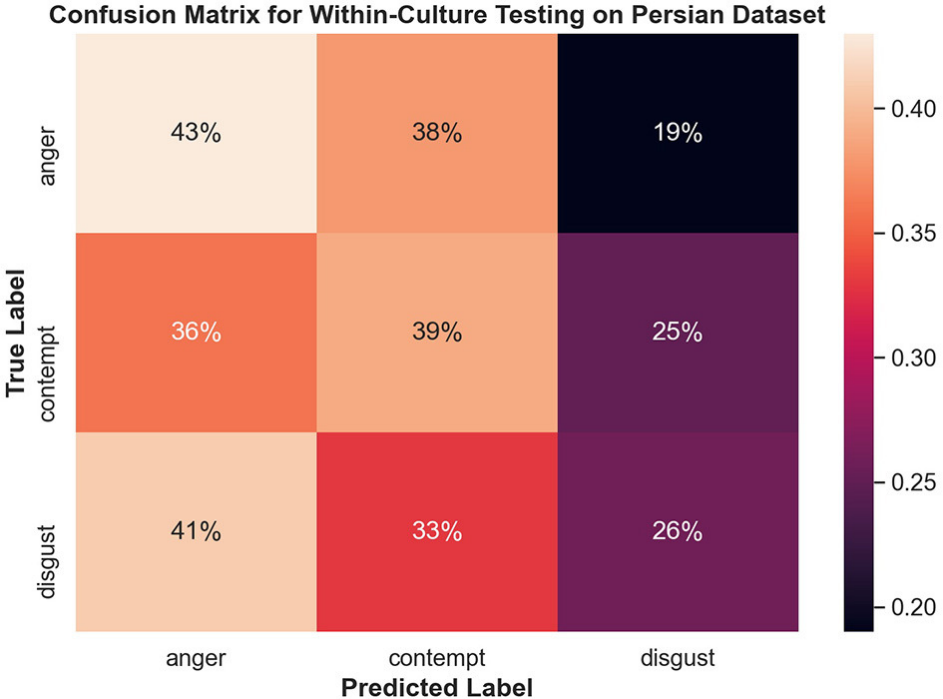
Confusion matrix illustrating the results of the within-culture experiment on the Persian data set.

**Figure 11:**
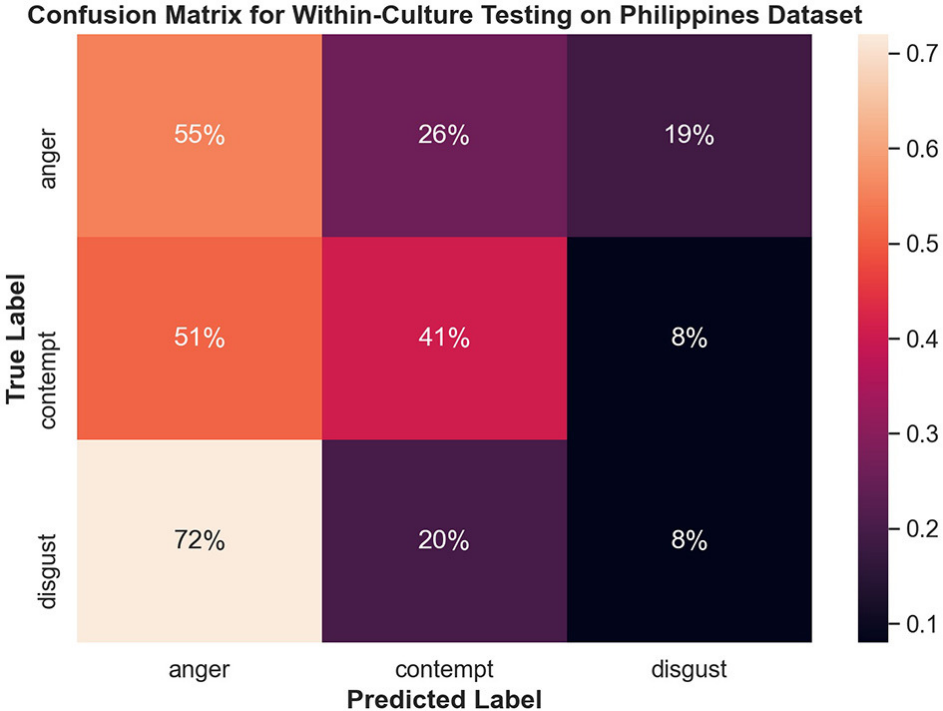
Confusion matrix illustrating the results of the within-culture experiment on the Filipino data set.

Both disgust and contempt for the within-culture experiment on the North American data set (Figure 9) had the highest accuracy with 72 and 69%, respectively. Anger performed the worst with 45% accuracy and was incorrectly classified as disgust 34% of the time. For within-culture experiment using the Persian data set (Figure 10), anger performed best with 43%. Anger was also confused 38% of the time with contempt. Disgust was commonly confused with anger, with 41% of the disgust clips being incorrectly labeled as anger and only 26% of disgust clips being correctly classified. On the other hand, contempt was split between either being correctly classified 39% of the time or misclassified, 36% of the time, with anger. Finally, the within-culture Filipino data set (Figure 11) performance was highest for anger with 55% accuracy. However, contempt and disgust were frequently mistaken for anger with 51% of contempt and 72% of disgust being mistaken as anger for the Filipino data set.

#### 3.4.3. Cross-Culture Confusions

The North American data set achieved the best test results in the cross-culture experiment with training on Persian with approximately 47% accuracy, while training on Filipino could reach approximately 36% accuracy. The Persian data set for the cross-culture experiment performed similarly to the within-culture experiment with 30% accuracy when trained on the Filipino data set and 40% accuracy when trained on the North American data set. When trained on the Persian data set, the Filipino data set resulted in a small increase of 40% accuracy, while 36% accuracy was present when trained on the North American data set for the cross-culture experiment.

## 4. Discussion

Fundamentally, the current study aimed to better understand the data being used to train emotion recognition models, as cultures are currently being treated equally in state of the art emotion recognition systems. In order to do so, we identified underlying differences in our cross-cultural data set, which focused here on negative emotion expressions. In recent years, it has become crucially important to recognize the impact culture has on developing rules to express oneself. Therefore, in order to increase emotion recognition performance one must consider culture. Our first hypothesis was supported using a one-way ANOVA to illustrate that each culture in our data set had significantly different means of activation for AUs related to CAD. As we saw from Figure 7, the level of activation for AUs associated with a given emotion significantly differed for each cultures. In particular, AUs related to the upper-half of the face, such AU4 (brow lowerer), significantly differed. Several of the AUs mentioned for each emotion significantly differed cross-culturally, the AUs prototypical to contempt and disgust were similar cross-culturally. More specifically, AU9 (nose wrinkler) did not significantly differ between Persian and Filipino and AU10 (upper lip raiser) did not significantly differ between North American and Filipino data sets. AU10 demonstrates a sneer, which is a social signal commonly attributed to contempt (Matsumoto and Ekman, 2004), whereas AU9 demonstrates a wrinkling of the nose, which commonly occurs in displays of disgust (Scherer et al., 2019; Singh et al., 2020). In addition, AU4 (brow lowerer), which is commonly seen amongst most negative emotions, did not significantly differ for disgust between the Filipino and North American data sets (Scherer et al., 2019).

The second hypothesis was only partially supported. The results of the SVM showed that the North American culture category performed the best on training and testing in the within-culture experiment compared to the cross-culture experiment. However, the collectivist cultures performed best when trained on the North American data set. Therefore, the cross-culture experiment performed better than the within-culture experiment on the collectivist data sets. As previously mentioned, several studies have hinted at a suppression of negative emotions in collectivist cultures (Ekman, 1971; Elfenbein and Ambady, 2002; Elfenbein et al., 2007). Thus the results could indicate that individualistic cultures allow automatic emotion recognition systems to be highly sensitive to emotion expressions as these cultures encourage stronger activation of AUs. If the collectivist cultures suppressed their emotions more this could indicate that the North American data set provided better training boundaries, giving a better training data set for the collectivist cultures.

In addition, contempt and disgust were commonly confused with anger for the Persian data set. One study by Elfenbein et al. (2007), found that for emotions of contempt and anger, facial muscles are activated similarly cross-culturally, whereas facial muscles related to disgust were found to significantly differ cross-culturally. This could explain why there was confusion with anger and could indicate similarities in AU activation across all three negative emotions, or that features were missing that were not included in the Persian data set, such as hand gestures.

For the Filipino data set, anger was commonly confused with contempt, while disgust was commonly confused with anger. This could mean that anger in the Filipino culture is similar to contempt and that the AU's expressed with disgust are similar to anger. Once again, however, this confusion could indicate that there are important features missing for the Filipino data set. The study by Elfenbein et al. (2007) also attributed that certain cultures depicted weaker displays of disgust, causing confusion with the cultures that displayed stronger depictions of disgust. As such, weaker displays of disgust could be why collectivist cultures performance in both experiments was low compared to the within-culture experiment of the North American data set.

Most interesting were the results obtained from the overall within-culture experiment. As the culture became more collectivist, performance accuracy decreased and obtained poorer recognition. This could indicate the social signals related to the CAD Triad slowly start to overlap and become similar. As mentioned, collectivist cultures tend to encourage suppression of negative emotions to maintain harmony. Stemming from environmental factors, collectivist cultures, such as Filipino and other East Asian and Southeast Asian cultures, value low arousal (or activation) of social signals (Lim, 2016; Schouten et al., 2020), enhancing the idea that collectivist cultures do not display emotions the same as individualistic cultures. Consequently, either the CAD Triad may have low levels of activation or the AUs associated with the CAD Triad become similar due to this suppression.

Together, the experiments show that the level of AU activation is different for each culture in our data set. Even though the AU might have been present for all cultures, this difference in AU activation supports the idea that the underlying components of negative emotional expressions are not universal. Therefore, training emotion recognition systems must consider culture in order to remove bias in the data set and potentially boost performance.

### 4.1. Explanatory Power of Selected Facial Action Units

The confusion matrices in Figure 8 illustrate the limitations of the SVM model with the proposed feature set, especially for Filipino and Persian samples. For instance, all Filipino disgust samples were identified as anger. One possible explanation is that, upon qualitative inspection, while AU4 (brow lowerer) is a common action unit between Filipino anger and disgust, disgust also includes an aversive gaze away (AU 51 or AU 52, not included in this study). Furthermore, a distinctive feature of prototypical and North American disgust is AU9 (nose wrinkler), but this was not present in Filipino disgust. One likely explanation was that the North American data contained more reality TV depictions of physical disgust (e.g., reactions to aversive food), whereas Persian and Filipino clips contained more moral disgust toward a person. This points to the importance of distinguishing sub-types of disgust when performing in the wild data collection. Adding gaze-related action units may thus improve distinction for Filipino disgust. Secondly, Persian anger was often mistaken for contempt in the SVM experiment, and this confusion is supported by anger and contempt's similar heat map activations in Figure 7. Qualitative interviews with a Persian annotator suggested that hand gesturing is an important social signal during these social displays, indicating that the speaker is referring to another person. Therefore, adding body pose or hand gestures is expected to improve performance.

### 4.2. The Relationship Between Kappa Values and Classification

As previously mentioned, the North American data set had the highest rate of agreement amongst annotators with a Fleiss Kappa value of 0.83. Both Persian and Filipino data sets had much lower rates of agreement, with Filipino having the lowest at 0.40 and Persian with 0.59. Furthermore, in terms of within culture classification accuracy, North American (66%) had the highest accuracy followed by Filipino (39%) and then by Persian (37%). Although not linear, the less agreement in the data set led to lower accuracy in classification. One reason for this low accuracy might be due to the suppression of social signals in the collectivist groups. As previously mentioned by Fernandez et al. (2000), Schouten et al. (2020), and Hofstede (2007), collectivist cultures suppress their negative emotions more to maintain harmony. Therefore, these cultures displays of negative emotions can look vastly different from the stereotypical North American display. This is also demonstrated in Figure 7, which displays the different level of AU's across all cultures for each emotion and clearly depicts the suppression of commonly seen AUs in collectivist cultures. As such, this might explain why there was lower agreement amongst annotators for the collectivist cultures. If collectivist cultures suppress commonly associated AUs then it becomes increasingly difficult to decipher what differences do exist between emotions in these cultures.

In summary, while the face has been the focus of many studies under the common view (resulting in relatively high accuracy for North American samples), our results suggest more investigation into non-facial features could help improve recognition of CAD emotions in non-Western cultures. Rather than considering that social signals are suppressed for collectivist cultures, it could rather be that *facial features are not enough*; important distinguishing information is communicated through other channels such as body pose and gesture as discussed here, voice (Tanaka et al., 2010), context (Ko et al., 2011), and so on.

### 4.3. Limitations

There were four main limitations of the current study. The first such limitation is that, while all data was collected “in-the-wild” outside of a laboratory using internet sources, analysis revealed that the data contained both professionally acted material (e.g., scripted TV) and spontaneous material (e.g., reality TV). A majority of the clips from the North American data set contained in-the-wild facial expressions collected from North American YouTube accounts which were “vlog” style content, reality television shows (e.g., Dance Moms), and talk shows (e.g., The Late show with James Corden). The Persian and Filipino data sets primarily contained data from YouTube accounts sharing clips from popular TV shows and movies from that area of the world. Furthermore, according to our Persian confederate, reality TV shows are uncommon in their culture. The result could point to the importance of distinguishing between in-the-wild acted vs. in-the-wild spontaneous videos, which currently is not considered during data collection in affective computing data sets. Consequently, we plan on diversifying the collectivist data sets with more realistic in-the-wild depictions in the future. Overall, this study attempted to move past solely acted depictions of Western expressions of contempt, anger and disgust, and this was achieved in this data set.

In addition to type of data collected, the data sets in general were relatively small. Each culture category had between 74 and 105 video clips, and amount was further reduced for each culture when considering each emotion separately. Although the data distribution for each emotion was similar for all cultures, more data could have increased the accuracy and f1-scores for each experiment. This is especially so for the Filipino data set which not only performed the worst in both experiments but also had the fewest number of overall video clips. In addition to the discrepancy for culture specific data set size, the overall accuracy was low. The current study only had 257 available video clips, which is relatively small. Other studies have used more advanced classification techniques to tackle the issue of small data sets. For example, Ma et al. (2019) used XGBoost to classify 1,323 video clips of to classify 6 different emotions. They found that XGBoost outperformed other classification methods, including an SVM. Future studies will explore the use of XGBoost in addition to increasing data set size in order to improve overall classification performance.

Another limitation is that image frames were used as input data, whereas videos were annotated. We cannot be certain that each individual image from a given video is depicting the labeled emotion. For example, a video labeled “contempt” could have one or more frames that do not display the emotion “contempt.” The results thus involve relative findings under these constraints. In the future, we will run a pretrained state-of-the-art AU detector on the data set and see what it results in, for a more absolute baseline. Future research can study the dynamics of negative emotions in culture by using a sequence of frames as input. It is also important to note that although the inter rater agreement was low for the Filipino data set, other studies on large affective image data sets have garnered similar agreement (Mollahosseini et al., 2019).

Finally, we had limited access to available AUs from OpenFace. There were several AUs that could not be collected that were important when describing prototypical emotions. For anger AU27 (Mouth Stretcher) was missing and AU20 (Lip Stretcher) was missing for disgust. Furthermore, contextual information could not be extracted from OpenFace. This contextual information includes hand gestures and audio information. Upon visual inspection of the Persian data set, hand gestures were commonly present. As such, future studies will look into extracting these contextual features and exploring other modes of detecting more AUs.

### 4.4. Future Studies

When we, as humans, perceive emotion, we do not only pay attention to facial expression but at the body as a whole (Aviezer et al., 2012). Hence, we cannot ignore the importance of body language such as head or hand movement. Adding data about body language in later research can lead us to testing more general hypotheses about role of culture in emotion. Moreover, it may help to build more accurate classifiers. Another opportunity to further investigate the role of culture is to provide more detailed annotations, such as extracting more social signals from videos. As such future work may use a method other than OpenFace to extract more AUs, since OpenFace is not capable of extracting some AUs such as AU27 (Mouth Stretcher), AU44 (Squinting) or AU24 (Lip Pressor) which we saw in our data set. Furthermore, potentially establishing the culture categories in an already published data set, such as AffectNet, could provide not only a larger data set but an already established ground truth. Currently, we are advancing methods in data augmentation to utilize advanced deep learning tools, like a Convolutional Neural Network, in order to boost recognition accuracy (Akhyani et al., 2021). Finally, having members of one culture annotate the labels of another culture could increase the evidence that cultures perceive emotions differently and present further underlying differences in certain social signals.

## Data Availability Statement

The original contributions presented in the study are included in the article/Supplementary Material, further inquiries can be directed to the corresponding author/s.

## Author Contributions

EH led and wrote the majority of the paper, implemented code for the experiments, and conducted data collection. RJ implemented code and performed data collection over the Persian sample. JT implemented the activation density map and also wrote parts of the methodology. AL wrote adding depth to the majority of the paper using her expert knowledge and provided supervisory oversight. All authors contributed to the article and approved the submitted version.

## Conflict of Interest

The authors declare that the research was conducted in the absence of any commercial or financial relationships that could be construed as a potential conflict of interest.

## Publisher's Note

All claims expressed in this article are solely those of the authors and do not necessarily represent those of their affiliated organizations, or those of the publisher, the editors and the reviewers. Any product that may be evaluated in this article, or claim that may be made by its manufacturer, is not guaranteed or endorsed by the publisher.
